# Mixed Bacillus Species Enhance the Innate Immune Response and Stress Tolerance in Yellow Perch Subjected to Hypoxia and Air-Exposure Stress

**DOI:** 10.1038/s41598-018-25269-z

**Published:** 2018-05-02

**Authors:** Nour Eissa, Han-Ping Wang, Hong Yao, ElSayed Abou-ElGheit

**Affiliations:** 1 Aquaculture Genetics and Breeding Laboratory, The Ohio State University South Centers, 1864 Shyville Road, Piketon, Ohio 45661 USA; 2 Department of Immunology, College of Medicine, University of Manitoba, Winnipeg, Canada; 3 Aquatic Diseases Laboratory, Aquaculture Division, National Institute of Oceanography and Fisheries, 101 Kaser El-Aini Street, Cairo, 11516 Egypt

## Abstract

Stress enhances the disease susceptibility in fish by altering the innate immune responses, which are essential defense mechanisms. The use of probiotics is increasingly popular in the aquaculture industry. Yellow perch is a promising candidate for aquaculture. We investigated the efficiency of a mixed Bacillus species in minimizing the potential problems resulting from husbandry practices such as hypoxia and exposure to air in yellow perch. We showed that hypoxia and air exposure conditions induced a significant reduction in the early innate immune response (lysozyme activity, *interferon*-*induced*-*GTP*-*binding protein*-*Mx1* [*mx*], *interleukin*-*1β* [*il1β*], *serum amyloid*-*A* [*saa*]), and a substantial increase in cortisol, heat shock protein (Hsp70), glutathione peroxidase (Gpx), superoxide dismutase (Sod1) that associated with a decline in insulin-like growth factor-1 (Igf1). Mixed Bacillus species administration improved the early innate responses, reduced cortisol, Hsp70, Gpx and Sod1, and elevated Igf1 levels. Bacillus species treated group showed faster recovery to reach the baseline levels during 24 h compared to untreated group. Therefore, mixed Bacillus species may enhance yellow perch welfare by improving the stress tolerance and early innate immune response to counterbalance the various husbandry stressors. Further studies are warranted to investigate the correlations between the aquaculture practices and disease resistance in yellow perch.

## Introduction

The innate immune system is a key defense missile of invertebrates and an essential defense mechanism of fish to maintain the homeostasis^1^. The early innate immune response in fish is regularly associated with the initiation of the inflammatory cascades such as interferon-induced GTP-binding protein-Mx1 (Mx), interleukin 1β (IL-1β), and serum amyloid-A (SAA)^2^ in response to a particular stressor^1,3^. Whereas Mx transcript is a direct marker of type I interferon (IFNα and IFNβ) activation, IL-1β is a proinflammatory cytokine, and SAA is acute phase protein primary synthesized in liver^4,5^. Moreover, the lysozyme triggers the complement system and phagocytic cells to fight against various pathogens^1^.

Probiotics can enhance the stress tolerance, immune responses and pathogen antagonism^6–9^. Stress is a contributing factor in the high mortality and diseases in aquaculture and fisheries stock enhancement^10^. Probiotics can enhance the stress tolerance in fish and other aquatic species by priming fish in advance to counteract environmental and husbandry stressors^11^, which sharply impact plasma cortisol levels in fish^12,13^. Cortisol is the primary corticosteroid hormone in fish and its levels are increased in response to various stressors^10,13^ to supplying the energy through glycogen, lipids and protein catabolism for metabolic adaptation^14^.

In fish, stress response occurs at the cellular level, which comprises various heat shock proteins (HSP), which have a protective role in maintaining the hemostasis^10^. Many studies reported a correspondence between increased levels of HSP and exposure to stressors, indicating that HSP are play a significant role in the survival and health of the stressed fish^15,16^. Stress has a negative impact on fish growth and the expression of insulin like growth factor (Igf). Stressors enhance the production of reactive oxygen species (ROS), which can endorse physiological dysregulation in fish due to high energy requirement and possible imbalance of the antioxidant defenses^17,18^. Fish possess antioxidant enzymes to counterbalance the cell damage and enzyme inactivation. These enzymes include superoxide dismutase (Sod1), which catalyzes the dismutation of superoxide radicals to hydrogen peroxide and oxygen. Glutathione peroxidase (Gpx) is a selenium-dependent enzyme, which crumbles peroxides using the peptide glutathione as their co-substrate that establish the antioxidant enzymatic defense^10^.

Yellow perch is a freshwater species that has economic importance in North America^13^. However, the physiological and immune responses of yellow perch have not been well studied and are poorly understood. Thus, we investigated the potential efficiency of commercial water-soluble probiotic product (Fishery Prime^TM^, Keeton Industries, USA), which is a mixed bacillus species, in the early innate immune responses and stress tolerance in yellow perch in response to hypoxia and exposure to air stress.

## Results

### A mixed Bacillus Species enhances the lysozyme activity in Yellow perch subjected to experimental hypoxia and exposure to air stressors

**Figure 1:**
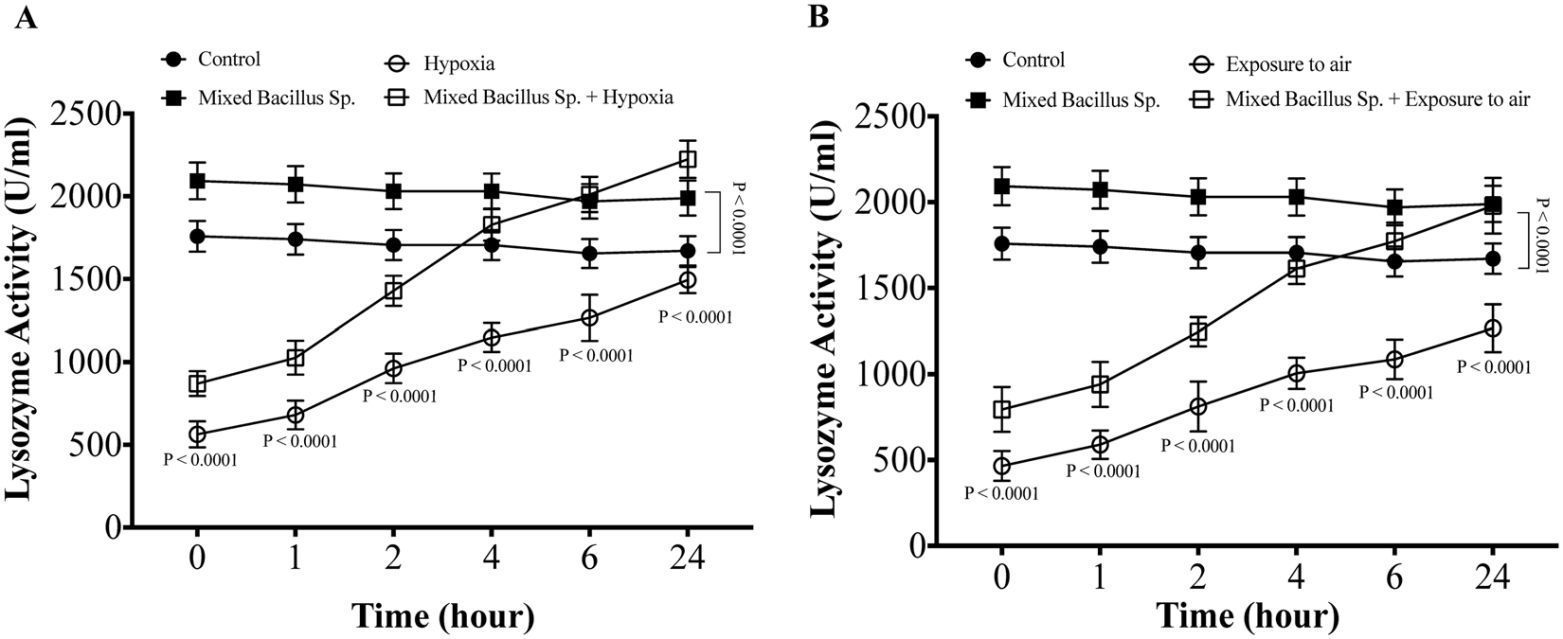
A mixed Bacillus Species enhances the lysozyme activity in Yellow perch subjected to experimental hypoxia and exposure to air stressors. Plasma lysozyme activity in Yellow Perch that subjected to (**A**) hypoxia and (**B**) air exposure stress over 24 h. Two-way Repeated measure ANOVA followed by comparing mixed Bacillus spp + stressed group to stressed groups was applied at two-tail significance level (P < 0.05). n = 9/group. Data represented in mean ± SD.

In naive conditions, the absence of stressors, the mixed bacillus sp. administration significantly elevated the plasma lysozyme activity compared to control group (Fig. 1A,B). Furthermore, yellow perch that subjected to experimental hypoxia and air exposure stressors showed a significant decrease in plasma lysozyme activity compared to the control groups (Fig. 1A,B). Yellow perch that subjected to experimental hypoxia and air exposure and received the mixed bacillus species showed a significant increase in plasma lysozyme activity compared to hypoxic and air exposed groups (Fig. 1A,B). Moreover, the mixed bacillus species administration accelerated the return of lysozyme to the proper range over the time within 24 h (Fig. 1A,B).

### A mixed Bacillus Species induces hepatic gene expression of early innate immune mediators in Yellow perch subjected to experimental hypoxia and exposure to air stressors

**Figure 2:**
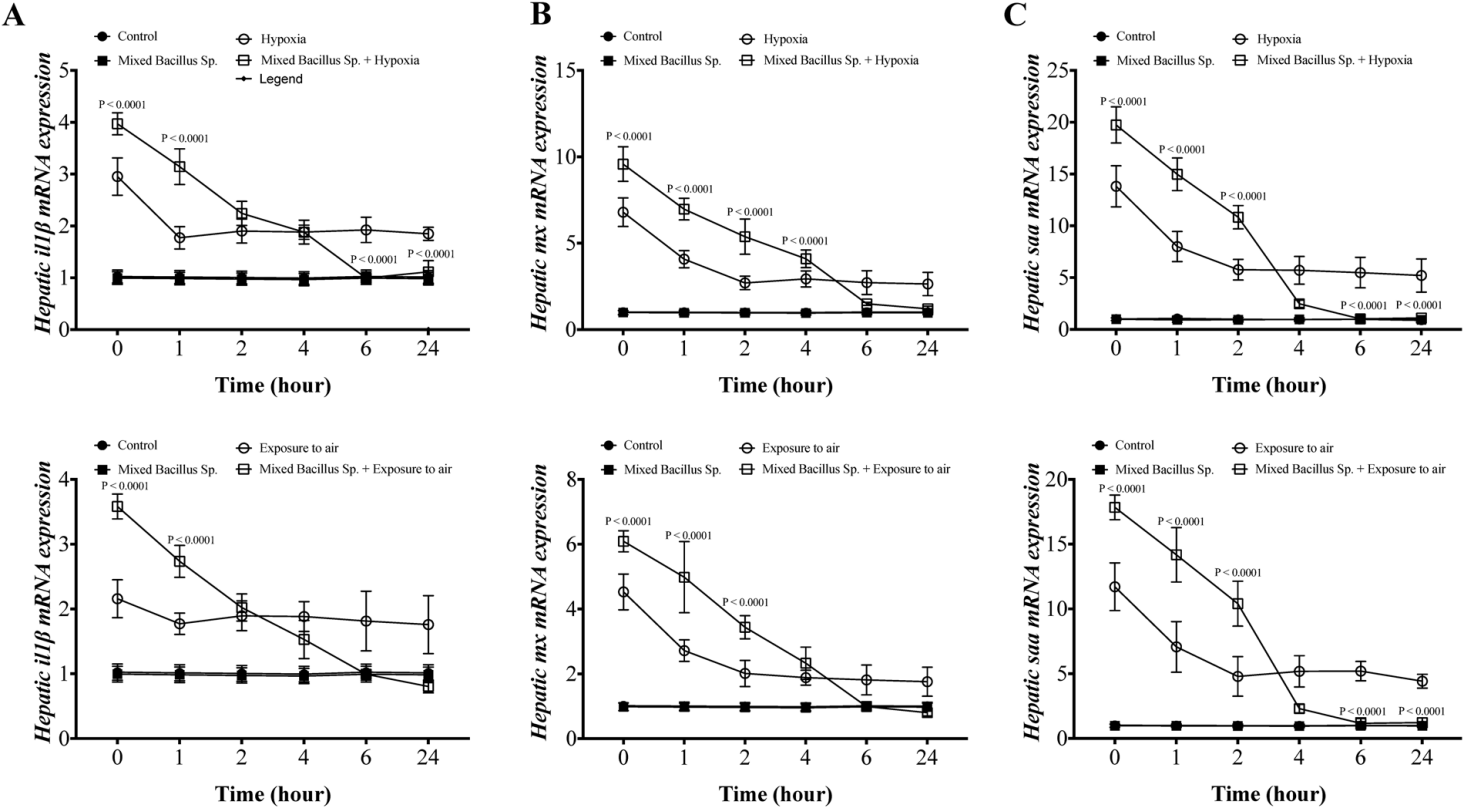
A mixed Bacillus Species induces hepatic gene expression of early innate immune mediators in Yellow perch response to experimental hypoxia and exposure to air stressors. Hepatic mRNA levels of (**A**) interleukin (*il1β*), (**B**) *mx transcript and* (**C**) *serum amyloid protein* (*saa*) in Yellow Perch that subjected to (**A**) hypoxia and (**B**) air exposure stress over 24 h. Sample size = 9/group. Two-way Repeated measure ANOVA followed by comparing mixed Bacillus spp + stressed group to stressed groups was applied at two-tail significance level (P < 0.05). Data represented in mean ± SD.

The experimental hypoxia and air exposure stressors in yellow perch resulted in a significant increase in hepatic gene expression of *il1β*, *mx*, and *saa* compared to the control groups (Fig. 2A–C). Interestingly, yellow perch that subjected to experimental hypoxia and air exposure and received the mixed bacillus sp. enhanced the increase of *il1β*, *mx* and *saa* levels compared to hypoxic and air exposed groups (Fig. 2A–C). Moreover, the mixed bacillus species administration accelerated the return of hepatic mRNA levels of *il1β*, *mx*, and *saa* to the normal range compared to untreated hypoxic and air exposed groups (Fig. 2A–C). In naive conditions, unstressed yellow perch, the mixed bacillus sp. did not have any significant effect on their levels (Fig. 2A–C).

### A mixed Bacillus Species attenuates cortisol-stress response in Yellow perch subjected to experimental hypoxia and exposure to air stressors

**Figure 3:**
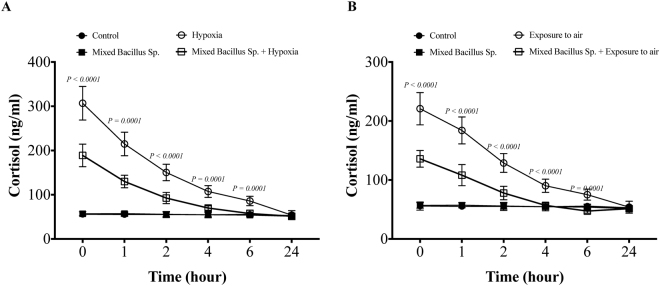
A mixed Bacillus Species attenuates the cortisol-stress response. Yellow Perch response to (**A**) hypoxia and (**B**) air exposure stress over 24 h as reflected in plasma cortisol levels. Sample size = 9/group. Two-way Repeated measure ANOVA followed by comparing mixed Bacillus spp + stressed group to stressed groups was applied at two-tail significance level (P < 0.05). Data represented in mean ± SD.

Yellow perch that subjected to experimental hypoxia and air exposure stressors showed a significant elevation of plasma cortisol levels compared to the control groups (Fig. 3A,B). Interestingly, yellow perch that subjected to experimental hypoxia and air exposure and received the mixed bacillus sp. showed a significant decrease in the plasma cortisol levels compared to hypoxic and air exposed groups (Fig. 3A,B). Moreover, the mixed bacillus species administration accelerated the return of cortisol levels to be within the normal range (Fig. 3A,B). In naive conditions, unstressed yellow perch, the mixed bacillus sp. did not have any significant effect on the cortisol levels (Fig. 3A,B).

### A mixed Bacillus Species promotes plasma and hepatic Igf1 expression in naive yellow perch and that subjected to experimental hypoxia and exposure to air stressors

**Figure 4:**
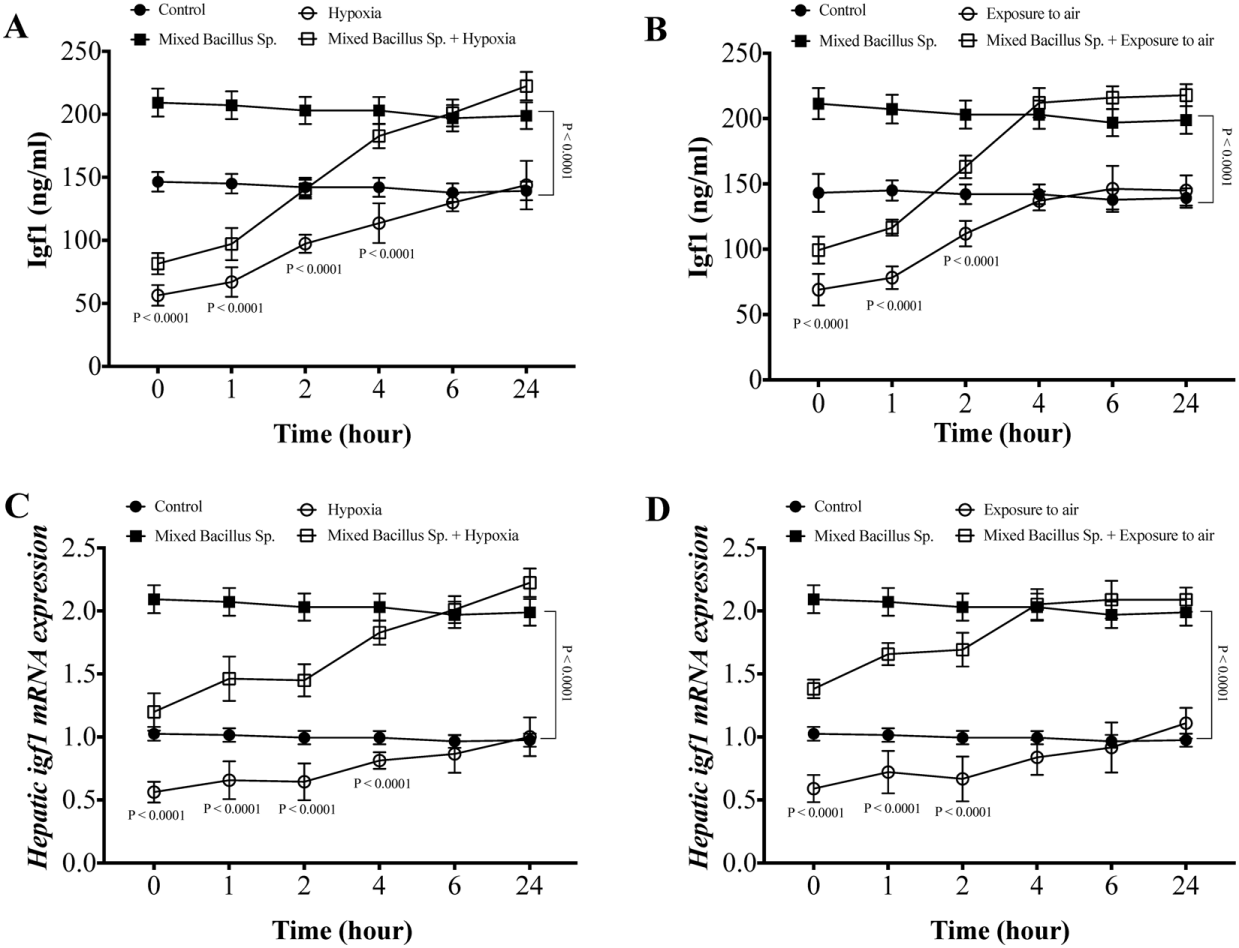
A mixed Bacillus Species promotes the expression of insulin like growth factor (Igf1). Yellow Perch response to hypoxia and air exposure stress over 24 h as reflected in (**A**,**B**) plasma Igf1 levels and hepatic *igf1* mRNA expression that were quantified using ELISA and RT-qPCR. Sample size = 9/group. Two-way Repeated measure ANOVA followed by comparing mixed Bacillus spp + stressed group to stressed groups and groups that received mixed Bacillus spp to groups that did not recieve was applied at two-tail significance level (P < 0.05). Data represented in mean ± SD.

In naive conditions, unstressed yellow perch, the mixed bacillus sp. administration significantly increased plasma Igf1 protein level and hepatic mRNA expression compared to control group (Fig. 4). Furthermore, yellow perch that subjected to experimental hypoxia and air exposure stressors showed a significant reduction not only in plasma Igf1 levels but also hepatic *igf1* mRNA expression compared to the control groups (Fig. 4). Remarkably, yellow perch that subjected to experimental hypoxia and air exposure and received the mixed bacillus sp. showed a significant increase in plasma protein level of Igf1 (Fig. 4A,B) and upregulation in hepatic *igf1* mRNA expression compared to hypoxic and air exposed groups (Fig. 4C,D). Moreover, the mixed bacillus species administration accelerated the elevation of Igf1 protein and mRNA levels to be within the normal range over the time-dependent manner (Fig. 4).

### A mixed Bacillus Species reduces plasma and hepatic Hsp70 response in Yellow perch subjected to experimental hypoxia and exposure to air stressors

**Figure 5:**
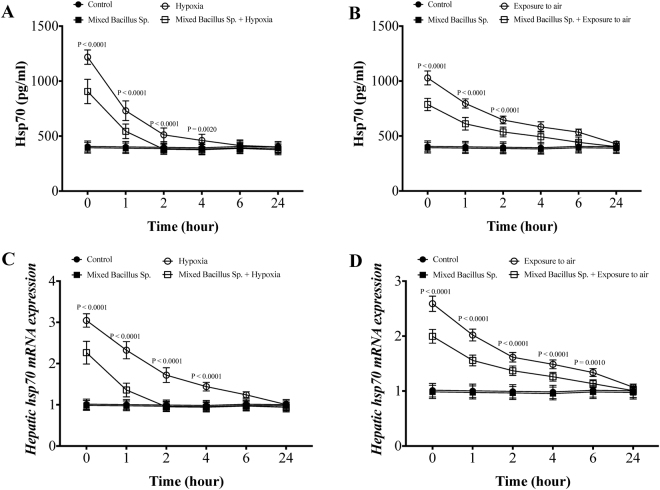
A mixed Bacillus Species decreases the expression of heat shock protein (Hsp70). Yellow Perch response to hypoxia and air exposure stress over 24 h as reflected in (**A**,**B**) plasma Hsp70 levels and hepatic *hsp70* mRNA expression that were quantified using ELISA and RT-qPCR. Sample size = 9/group. Two-way Repeated measure ANOVA followed by comparing mixed Bacillus spp + stressed group to stressed groups was applied at two-tail significance level (P < 0.05). Data represented in mean ± SD.

Yellow perch that subjected to experimental hypoxia and air exposure stressors showed a significant elevation of plasma Hsp70 protein level and hepatic *hsp70* mRNA expression compared to the control groups (Fig. 5). Fascinatingly, yellow perch that subjected to experimental hypoxia and air exposure and received the mixed bacillus sp. showed a significant decline in the plasma Hsp70 protein level and hepatic *hsp70* mRNA expression compared to hypoxic and air exposed groups (Fig. 5). Moreover, the mixed bacillus species administration accelerated the return of plasma Hsp70 protein levels and hepatic *hsp70* mRNA expression to be within the normal range over the time-dependent manner (Fig. 5). In naive conditions, unstressed yellow perch, the mixed bacillus sp. The probiotics did not have any significant effect on Hsp70 levels (Fig. 5).

### A mixed Bacillus Species decreases oxidative stress response in Yellow perch subjected to experimental hypoxia and exposure to air stressors

**Figure 6:**
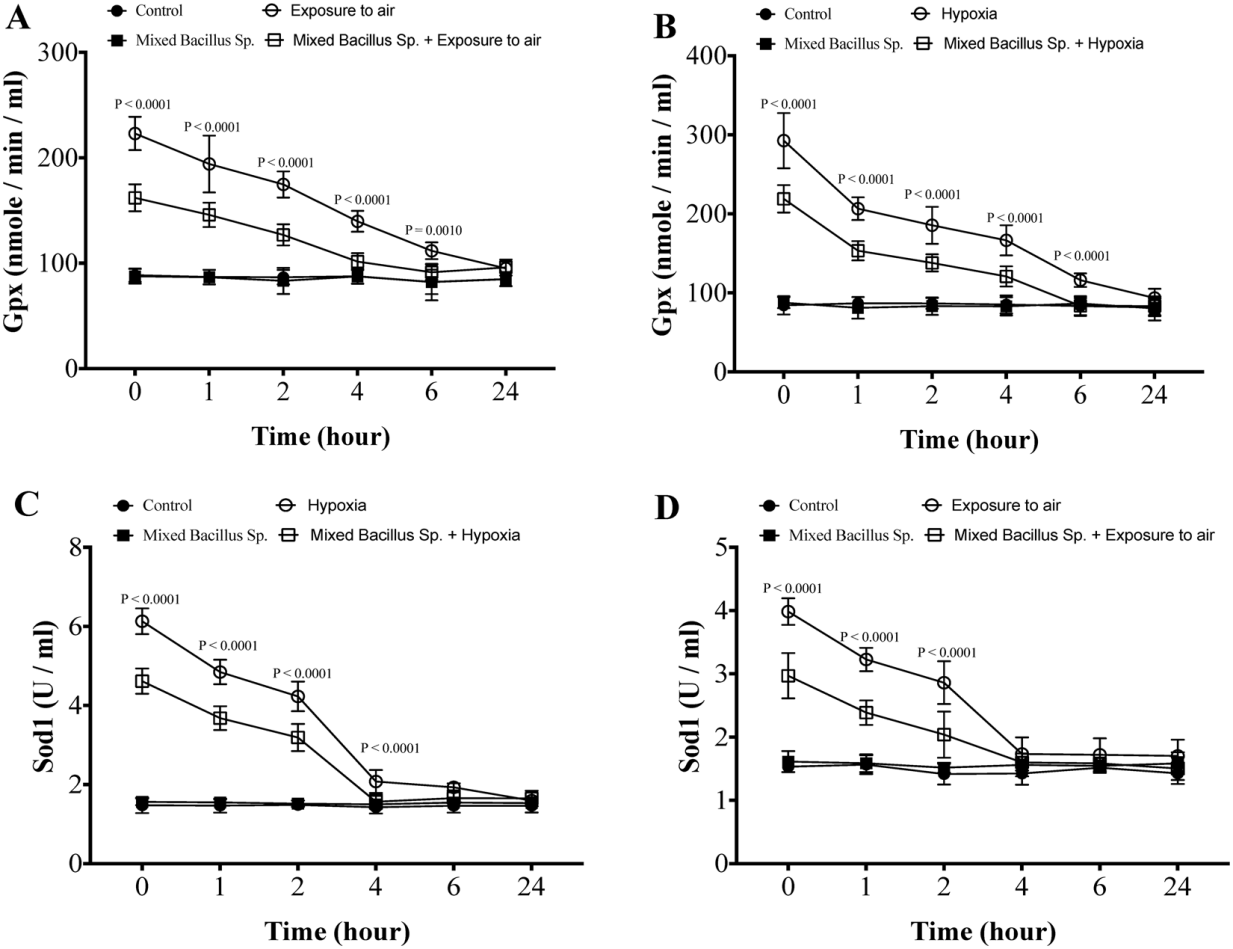
A mixed Bacillus Species lessens the plasma glutathione peroxidase (Gpx) and superoxide dismutase (Sod1). Yellow Perch response to hypoxia and air exposure stress over 24 h as reflected in plasma (**A**,**B**) Gpx and (**C**,**D**) Sod1 levels that were quantified using ELISA. Sample size = 9/group. Two-way Repeated measure ANOVA followed by comparing mixed Bacillus spp + stressed group to stressed groups was applied at two-tail significance level (P < 0.05). Data represented in mean ± SD.

**Figure 7:**
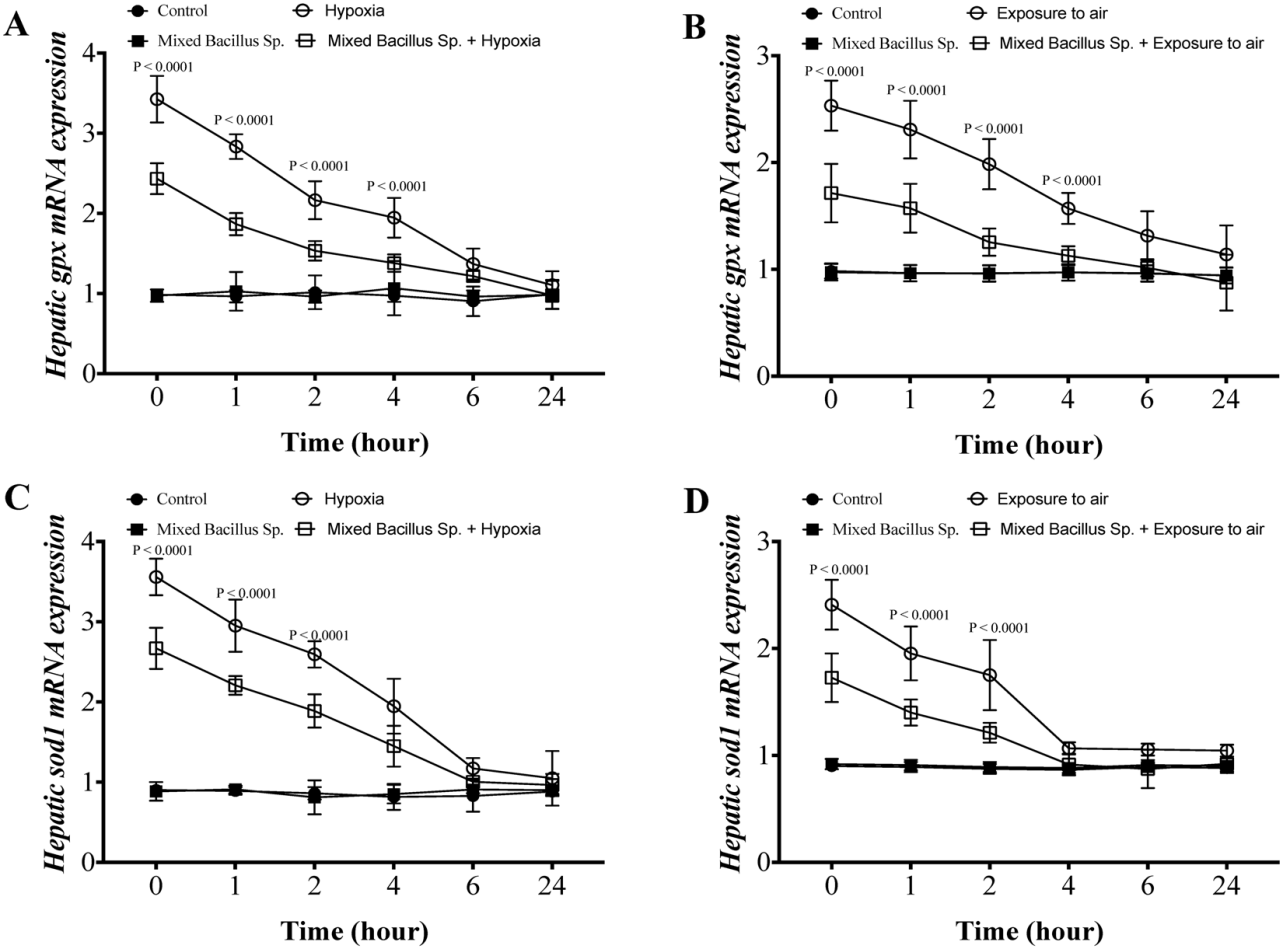
A mixed Bacillus Species reduces the hepatic glutathione peroxidase (*gpx*) and superoxide dismutase (*sod1*) gene expression. Yellow Perch response to hypoxia and air exposure stress over 24 h as reflected in hepatic mRNA levels of (**A**,**B**) *gpx3* and (**C**,**D**) *sod1* that were quantified using RT-qPCR. Sample size = 9/group. Two-way Repeated measure ANOVA followed by comparing mixed Bacillus spp + stressed group to stressed groups was applied at two-tail significance level (P < 0.05). Data represented in mean ± SD.

Yellow perch that subjected to experimental hypoxia and air exposure stressors showed a significant elevation of plasma Gpx and Sod1 protein levels as well as their hepatic mRNA expression compared to the control groups (Figs 6 and 7). Excitingly, yellow perch that subjected to experimental hypoxia and air exposure and received the mixed bacillus sp. showed a significant reduction in the plasma Gpx and Sod1 protein level and hepatic *gpx and sod1* mRNA expression compared to hypoxic and air exposed groups (Figs 6 and 7). Moreover, the mixed bacillus species administration accelerated the return of oxidative stress markers to be within the normal range over the time-dependent manner (Figs 6 and 7). In naive conditions, unstressed yellow perch, the mixed bacillus sp. The probiotics did not have any significant effect on Gpx and Sod1 levels (Figs 6 and 7).

The time effect, group effect and their interactions on all investigated parameters showed statistical significance (P < 0.0001).

## Discussion

The present study provides an evidence on the beneficial features of water-soluble mixed bacillus species on the physiological and molecular early innate immune and stress responses in yellow perch, which is an important aquaculture species in North America. This study showed that mixed bacillus species enhanced the lysozyme activity and the mediators of acute phase response that are critical as the first line of defense against various pathogens. Furthermore, the mixed bacillus species minimized the stress responses that exhibited a decrease in cortisol, Hsp70 signaling, and oxidative stress-related markers and an increase in the growth-related factor (Igf1). These consequences are beneficial, where aquaculture species could be treated in advance with probiotics to face critical hypoxia and exposure to air that weakened the immune defense mechanisms.

The innate immune system, including physical barriers, and cellular and humoral components, functions as a defense weapon in invertebrates^11^. Early innate immunity (lysozyme activity and acute phase inflammatory mediators [*il1β*, *mx*, *saa*]) is the first line of defense in fish and provides the initial resistance to pathogens within the first hours^2,19^. We found that mixed bacillus species, a probiotic, enhanced the early innate responses of yellow perch in response to hypoxia and air stress. Air exposure stress and hypoxia can result in several physiological and behavioral consequences of catch-and-release angling such as mucus removal, which is critical in the defense mechanism^20^. Therefore, our study show that mixed bacillus species can increase early innate immunity and compensate the mucus removal. Several reports also linked the beneficial effects of probiotics with the upregulation of immune related genes, which refer to appropriate functions of the innate immune response^8,9,21,22^. However, an aberrant immune activation results in detrimental effects that impact overall health^11,23–26^ supporting the potential immunomodulatory of mixed bacillus species by accelerating the recovery from the acute phase innate immune response in this study.

Cortisol plays critical roles in osmoregulation and to mitigate various infectious and non-infectious stressors^27–29^ by regulating the metabolic energy, hydromineral balance, oxygen uptake, hemostasis, and immune competence^14,30^. However, chronic increase in cortisol levels is harmful to fish^31^. Our study showed that mixed bacillus species reduced the plasma cortisol levels in yellow perch that subjected to hypoxic and air exposure stress. We showed previously that yellow perch subjected to handling and thermal stress elevated cortisol levels^13^. Probiotics attenuate the high cortisol levels in fish species subjected to different stressors^32,33^. These results can explain that the use of prophylaxis probiotics may elevate energy accessibility to supply with the metabolic support and expand the stress coping capability of fish^34^. Although we only investigated the cortisol levels but also sympathetic-chromaffin cells system plays central roles in the stress responses to establish the paracrine immune-endocrine interactions in fish^35^. Catecholamines secretion from fish chromaffin cells is mediated by a host of cholinergic and non-cholinergic pathways that warrant sufficient dynamics in the secretion process to permit harmonized responses to a wide array of stressors^36^. Therefore, the probiotics might be beneficial in mitigating the deleterious effects of various stressors by regulating hypothalamic-pituitary-interrenal (HPI) and sympathetic-chromaffin cells systems.

Cortisol is the primary hormonal reactions during the primary stage of the stress response^14,30^. However, Cortisol alone does not afford definitive explanations for the stress responses^14^. Thus, we investigated the plasma protein and hepatic mRNA expression levels of of Hsp70. Interestingly, we found that water-soluble mixed Bacillus species decreased the levels of Hsp70 significantly in time dependent manner. A characteristic feature of fish with stress response can be reflected by various Hsp changes^37,38^, which play important roles in stress physiology and endocrinology, stress tolerance, and acclimation^39^ by regulating glucocorticoid receptors^40,41^. IGF-I is extremely vital for the regulation of growth and cell functions^42^. Stress is reported to have severe negative effects on growth^43^. Our study showed that mixed bacillus species induced the release of plasma Igf1 and hepatic *igf1* mRNA levels in yellow perch that subjected to hypoxic and air exposure stress. Moreover, in naive condition, yellow perch that received mixed Bacillus spp. showed an elevated Igf1 level to compared to control group suggesting an increase in the growth performance parameters. Our results in accordance with that the administration of probiotics promotes Igf1 expression^44^ and correlates with the growth performance^45^. Stress induces oxidative tissue damage by enhancing the production of reactive oxygen species (ROS), which further promotes protein damage and activate the antioxidant system (Gpx and Sod1) to neutralize the impact of ROS^46^. Attractively, we found that water-soluble mixed Bacillus species diminished Gpx and Sod1 levels. The anti-oxidative properties of probiotics help to counteract oxidative stresses by promoting the early innate immune system^17^ as we showed in this study. A mixture of multi-species probiotic acts beneficially in mitigating the stressful effects in a tropical freshwater fish^46^. Furthermore, probiotics administration led to a significant decrease of Sod1 and *gpx* gene expression^17,47^.

This study is, up to our knowledge, the first example of the evaluation of beneficial effects of a mixed bacillus species on the kinetics of early innate immune and stress responses of yellow perch in response to hypoxia and exposure to air stressors. To cope with stress, fish need to be immunologically ready to meet the demand and quickly restore the normal physiological conditions. This can be achieved through preparing the fish in advance with probiotic treatment to mitigate the common aquaculture stressors. This study is a positive step to find more alternative ways to increase immune function and stress tolerance and improve yellow perch aquaculture. Further studies are required to understand how exactly mixed Bacillus spp enhanced the immune response and stress tolerance by investigating the involved intracellular signaling pathways.

## Material and Methods

### Animal ethics

The experiments were designed and conducted at the Aquaculture Research Center, The Ohio State University South Centers, Piketon, OH, USA. All experimental procedures involving animals were approved by the Ohio State University Institutional Animal Care and Use Committee and were carried out in compliance with the U.S. regulations governing biological research with vertebrates.

### Experimental Fish

Yellow Perch (20 ± 2.5 g) were obtained from the Aquaculture Research Center, Ohio State University South Centers, Ohio, USA. Before transfer, fish were grown together in 1000-L tanks and fed twice daily to satiation with a commercial diet, AquaMax^TM^ basal diet (Crude Protein Minimum 45–50.0%, Crude Fat Minimum 16.0%, Crude Fiber Maximum 3.0%, Phosphorous (P) Minimum 1.3%, Sodium (NA) Minimum 0.1%, Sodium (NA) Maximum 0.5%). Two weeks before experimentation, fish were transferred to twenty-four 400-L tanks (60 fish/tank) to acclimate to the experimental system that has a single bio-filter in a recirculating water system. Salinity was maintained near physiological optimum at 2–3% and the water temperature was kept at 20 °C^13^.

### Probiotic administration

After acclimation, one of the experimented group, three replicates, depended on the natural water supply and served as a control (19 ± 2.5 g). The other group, three replicates, received (20 ± 1.5 g) the commercial mixed Bacillus species probiotic (Fishery Prime^TM^, Keeton Industries, USA) which is water soluble beneficial microbes’ product (Non-pathogenic naturally occurring bacteria on a food-grade cornmeal base Bacillus Species (*Bacillus subtilis*, *Bacillus pumlis*, *Bacillus amyloliqueficiens* and *Bacillus licheniformis*) and 70% water solubility). The probiotic applied directly into the water at rate 5 g daily for the Probiotic-Treated group for six weeks.

### Experimental design

#### Acute hypoxia

Air supply and water flow were stopped to three tanks from the probiotic receiving group and three tanks from the control group (An interval of 30-min between the tanks). Oxygen levels were monitored using a YSI-55 dissolved oxygen meter (YSI Inc., Yellow Springs, Ohio). After 5 h, when dissolved oxygen levels decreased to 1 mg/L, random three fish per replicate (9/group) were collected for blood and tissue sampling. The remaining fish were transferred into new, oxygenated tanks. Fish samples were collected at 0, 1, 2, 4, 6 and 24 h post-exposure to air.

#### Exposure to Air

Fish groups were subjected to air exposure stress by netting fish out of the tank for 60 seconds, and three fish per replicate were collected for further analysis (n = 9 fish/group) (with 30-min intervals between tanks). Fish samples were collected at 0, 1, 2, 4, 6 and 24 h post-exposure to air.

### Blood Plasma and tissue sampling

Fish were gently captured and immediately immersed in a bucket containing a lethal dose of tricaine methanesulfonate (400 mg/L) (Syndel Laboratories Ltd., Vancouver, British Columbia). Blood samples were collected from the caudal vein using a 5-ml heparinized syringe. Plasma was separated by centrifugation (1000 g for 10 min) at 4 °C, removed and stored in 1.5-ml microcentrifuge tubes at −80 °C for subsequent analysis. Fish were carefully dissected, and whole livers were collected.

### Plasma Lysozyme activity

Lysozyme activity was quantified using Lysozyme Detection kit’s (Sigma-Aldrich, USA) as described in the manufacture’s protocol. Each blood plasma sample was measured with three parallels, and their average was used for the analysis. Briefly, lysozyme activity was measured using a Microplate Spectrophotometer (BioTek’s Epoch™, USA) at 450 nm and using the following formula: Lysozyme activity (U/ml) = [(ΔA_450_/min Test − ΔA_450_/min Blank) × (dilution factor)]/[(0.001) × (0.03)].

### Cortisol and protein Assays

Plasma cortisol levels were determined using an enzyme-linked immunosorbent assay (ELISA) kit (NEOGEN^®^, Lexington, Kentucky) according to the manufacturer’s instructions. Briefly, 50 μL of standards or samples were added to the appropriate wells in duplicate, and then 50 μL of the diluted enzyme conjugate was added to each well and mixed by shaking plate gently then incubated for 1 h then washing. 150 μL of substrate were added and incubated at room temperature for 30 minutes. Plate was gently shaked before taking a reading to ensure uniform color throughout each well. Plates were read with BioTek microplate reader at an absorbance of 490–630 nm. Finally, absorbance values were calculated for standards and samples and the concentration of each sample was determined using the standard curve. The protein levels of Hsp70, Igf1, Gpx and Sod1^48^ in plasma were measured spectrophotometrically (BioTek’s Epoch™, USA) using colorimetric kits (Cayman Chemical, USA) and (MyBioSource, Inc. San Diego, CA, USA)^48^.

### Gene expression analysis

Total RNA was extracted from liver samples using following the TRIzol Reagent (Invitrogen, USA) protocol. The quality and quantity of RNA were assessed. Synthesis of cDNA was carried out using a High Capacity cDNA Reverse Transcription Kit (Applied Biosystem^®^, USA) following the manufacturer’s instruction in a Bio-Rad^®^ Thermo cycler. A quantitative real-time polymerase chain reaction (RT-qPCR) was performed using the real-time PCR 7500 system (Applied Biosystem®, USA) to quantify *il1β*, *mx*, *saa*, *hsp70*, *igf1*, *gpx3*, *sod1 and* β-actin mRNA expressions. The sequence of primers was used as published previously^2,49^ (Table 1). Primers were tested and validated using NCBI BLAST and Integrated DNA Technologies’ Oligoanalyzer 3.1. PCR efficiencies of the primers were between 90 and 100%. PCR amplifications were performed for each primer, and then the products were run on 1% agarose gels stained with SYBR^®^ safe (Invitrogen™, USA) to confirm that the primers amplified single products. Each amplification reaction mixture (20 μl) contained 100 ng of cDNA; 1× SYBR® Green Master Mixes (Applied biosystem, USA); 200 nM of each primer. The quantitative gene expression levels were calculated and analyzed^50,51^. All measurements were performed in triplicate. The specific quantities were normalized against the amount of β-actin amplified.

**Table 1:** Primers for genes in *Perca flavescens*.

Target gene	Primer	Sequence (5′–3′)	PCR product length (base pairs)	NCBI Accession #
*gpx3*	Forward	TGACTACACGGGCAAGAGTG	128	FJ826525.1
	Reverse	GGAAGCCAAGAAGGGTGAG		
*igf1*	Forward	CGC AGG GCA CAA AGT GGA C	102	AY332492.2
	Reverse	CCC AGT GTT GCC TCG ACT TG		
*sod1*	Forward	GCATGTAGGAGACTTGGGCAAT	64	KT783483.1
	Reverse	CCGTGATTTCTATCTTGGCAACA		
*hsp70*	Forward	TGTTGGTCGGTGGCTCAA	60	KX050165.1
	Reverse	TTGAAGAAGTCCTGAAGCAGCTT		
*β*-*actin*	Forward	GCCTCTCTGTCCACCTTCCA	62	AY332493.2
	Reverse	GGGCCGGACTCATCGTACT		
*il1β*	Forward	ATCTTGAGGTTGTGGAGGCA	176	GO656767.1
	Reverse	GCACATTTCCACTGGCTTGT		
*saa*	Forward	ACCATGCTCGTTTGCCTTCT	209	KC306947
	Reverse	TGTGGCGAGCATACAGTG AT		
*mx*	Forward	AAGAGGCAGTGGCATTGT	244	GO654167.1
	Reverse	AATGAGCGTCAGGTCTGGAA		

### Statistical analysis

Two-Way repeated measure analysis of variance (ANOVA) followed by multiple comparison tests was used for testing mean differences between groups at the different time points at significance levels (P ≤ 0.05). GraphPad Prism version 6 was used for all statistical analysis & creating the graphs.
